# Effects of season and artificial photoperiod on semen and seminal plasma characteristics in bucks of two goat breeds maintained in a semen collection center

**DOI:** 10.14202/vetworld.2017.521-525

**Published:** 2017-05-13

**Authors:** Francisco Arrebola, José-Alfonso Abecia

**Affiliations:** 1 Agriculture, Livestock and Fisheries Research Institute (IFAPA) Hinojosa del Duque, Carretera el Viso, km 2, 14270 Córdoba, Spain; 2 Environmental Science Research Institute of Aragon (IUCA), University of Zaragoza, Faculty of Veterinary Sciences, Miguel Servet, 177, Zaragoza, Spain

**Keywords:** buck, goat, semen, seminal plasma

## Abstract

**Aim:**

This study quantified the effects of season and photoperiodic treatment on semen and seminal plasma (SP) characteristics in 12 bucks of two Spanish goat breeds (Murciano-Granadina, and Payoya) for the past 1 year.

**Materials and Methods:**

A total of 6 bucks (three of each breed) were exposed to the natural day length and the other six males (three of each breed) were exposed to alternating conditions of 2 months of long days (16 h light) and 2 months of short days (8 h light). Weekly concentrations of glutamic oxaloacetic transaminase/aspartate aminotransferase (GOT/AST), lactate dehydrogenase (LDH), potassium, testosterone, and protein in SP were measured. Reaction time and scrotal circumference were recorded, and plasma testosterone concentrations were measured before semen collection.

**Results:**

Sperm volume, LDH, and potassium concentration in SP, and reaction time did not differ significantly between breeds, seasons, and photoperiodic treatment. Sperm concentrations were higher (p<0.001) in spring and summer than they were in autumn and winter. Mean percentage of positive hypo-osmotic swelling test sperm was the highest in summer and under the artificial photoperiod (p<0.01). GOT/AST concentrations differed (p<0.01) between breeds and seasons. Breed, season, and photoperiod had significant (p<0.001) effects on protein and testosterone levels in SP. Plasma testosterone concentrations were highest in summer (p<0.001), and differed significantly (p<0.01) between breeds. Scrotal perimeter differed significantly (p<0.001) between breeds and photoperiod.

**Conclusion:**

Recognition of those seasonal and breed-specific differences in the performance of bucks should help to improve the management of individual semen samples for use in artificial insemination programs.

## Introduction

Photoperiod controls sexual activity and is the main environmental factor that causes seasonality in reproduction in goats. Although seasonality is less marked in the buck than it is in the doe, males exhibit a pronounced seasonal reduction in sexual behavior and spermatogenesis at about the same time of the year as when females are in sexual rest, but with 1-2-month advance in phase [1].

Semen comprises spermatozoa suspended in seminal plasma (SP), a complex fluid medium that facilitates the physiological function of the ejaculate. SP includes, for example, ions, energy substrates, organic compounds, peptides, proteins, lipid, hormones, and cytokines [2,3]. Factors such as time of year, temperature, nutrition, and stress affect the protein content of the SP in the ram [4-6]. Probably, seasonal variation in gonadotropin levels and their receptors in the testes contributed to those seasonal differences [7]. Elsewhere, after detecting the presence of melatonin in the SP of the ram [8], it was concluded that melatonin is involved in the regulation of semen quality and the antioxidant enzyme activity that affect the reproductive performance of rams, and that seasonal variations of fertility in the ram involve an interplay between melatonin and the antioxidant defense system. Moreover, melatonin treatment during the non-reproductive season had an effect on its own levels in ram SP, and on some of the enzymes involved in the antioxidant defense system [9]. In the goat, the proteins in SP fluctuate seasonally and are involved in sperm function in the breeding and non-breeding seasons [10,11]. Moreover, both management system and season are factors affecting semen freezability and hence that semen collected from bucks reared under the semi-intensive system and winter season showed better semen freezability characteristics in India [12].

The use of artificial photoperiod treatments as a means of optimizing the production of semen for artificial insemination (AI) has been proposed. In Mexico, alternations between periods of long- and short-day length eliminated seasonal variations in the quantitative and qualitative characteristics of bucks [13] and abolished the seasonal variation in semen quality without altering sperm fertility [1]. Furthermore, artificial changes in photoperiod allow the production of AI doses year round under large-scale field conditions [14]. This strategy has also been applied for depositing high-quality sperm in genetic resource banks, beyond the normal breeding season from wild goat species [15].

The aim of this study was to characterize the SP of two native goat breeds in Spain, Murciano-Granadina (MG) and Payoya (P), which were maintained under either natural or artificial photoperiods. Although seasonality in the buck has been described, most studies have focused on sperm volume and concentration, and few have examined the effects of season on SP composition. This study examined the effects of season, breed, and photoperiodic treatment on semen and SP in bucks maintained in a semen collection center.

## Materials and Methods

### Ethical approval

The care and use of animals were in accordance with the Spanish Policy for Animal Protection RD1201/05, which meets the European Union Directive 2010/63 on the protection of animals used for experimental and other scientific purposes.

### Sample collection

For 1 year, 12 adult bucks (2-5 years) of two Spanish goat breeds (MG: n=6; P: n=6) were housed individually at the IFAPA Research Centre of the Andalusian Government (Hinojosa del Duque, Cordoba, Spain, 38°N). Six bucks (three of each breed) were housed in barns that had a concrete floor and large skylights and had access to external paddocks, which exposed them to the natural day length. The other six males (three of each breed) were kept in a light-controlled environment and exposed to alternating conditions of 2 months of long days (16 h light) and 2 months of short days (8 h light). Groups were balanced for live weight and age. Semen was collected at weekly intervals at 0800 h for 12 months, placing a teaser doe in a restraining bail, introducing the buck to the teaser and collecting the ejaculate in an artificial vagina.

### Semen and SP analysis

Semen production was assessed based on ejaculate volume, which was estimated by weighing the semen sample in the collection vessel immediately after it was collected [16], and sperm concentration, which was measured using a spectrophotometer (Accucell, IMV, France) that was calibrated for goat semen (dilution 1:200). SP was separated from ejaculates by centrifugation at 5000 rpm for 10 min. The recovered SP fraction was further centrifuged at 10,000 rpm for 15 min at 4°C, and the supernatant was stored at −20°C. The reaction time of the buck was based on ejaculation latency (the time between the first contact with the female and ejaculation) [17]. Scrotal circumference was measured, and blood samples for measuring testosterone levels were collected just before semen samples were collected, by jugular venipuncture and placed into heparinized tubes; samples were centrifuged at 3500×*g* for 30 min, and the plasma fraction was stored at −20°C until assay. Glutamic oxaloacetic transaminase/aspartate aminotransferase (GOT/AST), lactate dehydrogenase (LDH), and protein concentrations in goat SP were measured using atomic absorption spectrometry (GBC 906, AA, USA). Potassium (K) concentrations were measured by indirect potentiometry (Synchron System, Beckman, USA). Testosterone concentrations were measured by chemiluminescence (Immulite Immunoassay System, Siemens Healthcare, Munich, Germany); inter- and intra-assay coefficients of variation were 9.3% and 10.1%, respectively. Plasma testosterone concentrations were measured in duplicate by a radioimmunoassay using a commercial assay kit (Siemens, México), which had a sensitivity of 0.4 ng/ml; inter- and intra-assay coefficients of variation were 6.7% and 8.0%, respectively.

Hypo-osmotic swelling test (HOST) for the evaluation of sperm membrane integrity was performed following methods previously described [18]. In brief, 10 ml of the sample were mixed with 1 ml of a hypo-osmotic solution and incubating at 37°C for 30 min. Only those sperm having a curling tail were considered HOS test–positive. 200 cells were evaluated by counting in at least 5 fields under a phase contrast microscope at 400× magnification.

### Statistical analysis

All the parameters under study were subjected to analysis of variance using a mixed model that included the fixed effects of breed (MG vs. P) season (spring, summer, autumn, or winter), and photoperiod regime (natural or artificial). To decrease the complexity of the statistical analyses, the ANOVAs were conducted in sequential stages. The first stage of analysis considered all of the main effects, and the second stage included all of the factors with significant main effects from the first stage plus all first-order interactions. The final ANOVA included the same main effects as the second-stage analysis plus all significant first-order interactions from the second stage and their interactions. Least significant difference post-hoc tests were used to identify statistically significant differences between groups. The results were expressed as a mean±standard error of the mean. The probability level for statistical significance was set at p<0.05.

## Results

Ejaculate volumes, LDH and K concentrations in SP and reaction times did not differ significantly between breeds, seasons, or photoperiod) (Table-1). Sperm concentrations did not differ between breeds or photoperiod treatment but were significantly (p<0.001) higher in spring and summer ejaculates than they were in autumn or winter ejaculates (Table-2). Mean percentage of sperm that was HOST test positive was highest in summer (p<0.01) and under the artificial photoperiod (p<0.01) (Table-3), and in summer, MG bucks had a higher (p<0.01) percentage than did P bucks. Breed and season had significant (p<0.01) effects on SP GOT/AST concentrations (Table-1), which were highest in the MG breed (Table-4), and in autumn and winter (Table-2). Breed, season, and photoperiod had significant effects on protein and testosterone levels in SP (Table-1); specifically, protein concentrations were highest (p<0.001) in winter ejaculates (Table-2), in MG bucks (p<0.05) (Table-4), and in bucks kept under the natural photoperiod (p<0.001) (Table-3), with significant differences between treatments in spring, summer, and autumn. Plasma testosterone concentrations were highest in summer (p<0.001) (Table-2), and significantly (p<0.01) higher in MG than they were in P bucks (Table-4).

**Table-1:** Statistical significance in the factorial model of each of the factors that might affect semen and SP characteristics, and plasma testosterone concentrations, reaction time to artificial vagina, and scrotal perimeter of MG and P bucks maintained under natural or artificial (2 months long days -16 h light, 2 months short days - 8 h light) photoperiod throughout the year.

Parameter	Model	Breed	Season	Photoperiod
Volume	NS	NS	NS	NS
Concentration	p<0.01	NS	p<0.001	NS
HOST	p<0.01	NS	p<0.01	p<0.01
GOT/AST	p<0.001	p<0.001	p<0.001	NS
LDH	NS	NS	NS	NS
K	p<0.001	NS	NS	NS
Protein	p<0.001	p<0.01	p<0.05	p<0.05
SP testosterone	p<0.001	p<0.05	p<0.001	p<0.001
Plasma testosterone	p<0.001	p<0.01	p<0.001	NS
Reaction time	NS	NS	NS	NS
Scrotal perimeter	p<0.001	p<0.001	NS	p<0.001

HOST test=Hypo-osmotic swelling test for the evaluation of sperm membrane integrity, GOT/AST=Glutamic oxaloacetic transaminase/aspartate amino transferase, LDH=Lactate dehydrogenase, K=Potassium, NS=Non significant, SP=Seminal plasma, MG=Murciano-Granadina, P=Payoya

**Table-2:** Seasonal variation (mean±SEM) in semen and SP characteristics, and plasma testosterone concentrations, reaction time, and scrotal perimeter of MG and P bucks maintained under natural or artificial (2 months long days - 16 h light, 2 months short days - 8 h light) photoperiod throughout the year.

Parameter	Spring	Summer	Autumn	Winter
Volume (ml)	1.23±0-06	1.77±0.62	0.94±0.06	1.12±0.05
Concentration (sperm×10^9^)	5.43±0.01^a^	5.39±0.13^a^	4.62±0.19^b^	4.82±0.14^b^
HOST (%)	40.63±2.61^a^	54.38±3.32^b^	48.47±3.12	42.21±3.09^a^
GOT/AST (uKat/L)	17.64±1.81^ad^	23.48±1.79^bd^	30.16±1.45^c^	29.02±2.45^c^
LDH (uKat/L)	57.15±1.81	60.48±2.85	71.20±2.34	69.44±5.74
K (mmol/L)	38.47±1.31	40.34±1.78	39.95±1.29	43.09±2.20
Protein (g/L)	38.27±1.64^a^	41.00±1.92	41.25±1.50	44.37±2.05^b^
SP testosterone (nmol/L)	31.30±2.24^a^	34.10±2.68^a^	35.90±2.30^a^	51.01±5.23^b^
Plasma testosterone (nmol/L)	4.49±0.72^ac^	10.60±1.19^bd^	3.58±0.53^c^	2.14±0.19^d^
Reaction time (s)	57.59±4.42	53.69±3.50	74.72±9.09	55.06±5.71
Scrotal perimeter (cm)	27.44±0.27	28.23±0.24	27.29±0.37	27.34±0.43

a,b,c,d Significant differences at least p<0.05. HOST test=Hypo-osmotic swelling test for evaluation of sperm membrane integrity, GOT/AST=Glutamic oxaloacetic transaminase/aspartate amino transferase, LDH=Lactate dehydrogenase, K=Potassium, SP=Seminal plasma, SEM=Standard error of mean, MG=Murciano-Granadina, P=Payoya

**Table-3:** Photoperiodic differences (mean±SEM) in semen and SP characteristics, and plasma testosterone concentrations, reaction time, and scrotal perimeter of MG and P bucks maintained under natural or artificial (2 months long days - 16 h light, 2 months short days - 8 h light) photoperiods throughout the year.

Parameter	Natural	Artificial
Volume (ml)	1.21±0.06	1.37±0.26
Concentration (sperm×10^9^)	5.20±0.11	5.16±0.09
HOST (%)	44.18±2.18^a^	48.20±2.27^b^
GOT/AST (uKat/L)	25.31±1.40	24.86±1.36
LDH (uKat/L)	64.30±2.47	65.17±2.30
K (mmol/L)	43.34±1.12	36.97±0.99
Protein (g/L)	44.79±1.29^a^	37.02±1.05^b^
SP testosterone (nmol/L)	40.75±2.06^a^	32.90±2.17^b^
Plasma testosterone (nmol/L)	5.57±0.83	4.98±0.51
Reaction time (s)	60.17±5.69	57.73±2.83
Scrotal perimeter (cm)	28.56±0.27^a^	27.17±0.20^b^

a,b Significant differences at least p<0.05. HOST test=Hypo-osmotic swelling test for evaluation of sperm membrane integrity, GOT/AST=Glutamic oxaloacetic transaminase/aspartate amino transferase, LDH=Lactate dehydrogenase, K=Potassium, SP=Seminal plasma, SEM=Standard error of mean, MG=Murciano-Granadina, P=Payoya

**Table-4:** Breed differences (mean±SEM) in semen and SP characteristics, and plasma testosterone concentrations, reaction time, and scrotal perimeter of MG and P bucks maintained under natural or artificial (2 months long days - 16 h light, 2 months short days -8 h light) photoperiod throughout the year.

Parameter	MG	P
Volume (ml)	2.10±0.03	1.69±0.39
Concentration (sperm×10^9^)	4.99±0.01	5.37±0.10
HOST (%)	48.07±2.47	44.32±1.96
GOT/AST (uKat/L)	29.41±1.36^a^	20.76±1.25^b^
LDH (uKat/L)	65.15±2.00	64.39±2.71
K (mmol/L)	40.48±0.87	39.84±1.30
Protein (g/L)	42.98±1.14^a^	38.83±1.30^b^
SP testosterone (nmol/L)	40.15±1.58^a^	33.49±2.57^b^
Plasma testosterone (nmol/L)	5.97±0.59^a^	4.03±0.62^b^
Reaction time (s)	65.87±3.63	50.19±3.62
Scrotal perimeter (cm)	26.33±0.20^a^	28.93±0.22^b^

a,b,c,d Significant differences at least p<0.05. HOST test=Hypo-osmotic swelling test for evaluation of sperm membrane integrity, GOT/AST=Glutamic oxaloacetic transaminase/aspartate amino transferase, LDH=Lactate dehydrogenase, K=Potassium, SP=Seminal plasma, SEM=Standard error of mean, MG=Murciano-Granadina, P=Payoya

Scrotal perimeter differed significantly (p<0.001) between breeds (Table-4) and photoperiod treatments (p<0.001) (Table-3); specifically, P bucks had the longest perimeters throughout in all seasons, and under the natural photoperiod in spring and autumn.

## Discussion

Many factors including breed, body weight, age, management, weather, nutrition (feed quality and availability), method of semen collection, and degree of sexual stimulation affect semen characteristics of bucks [19]. In this study, the potential effects of each of those factors were minimized because the groups were balanced by age and weight, bucks were kept in individual pens, and fed the same diet throughout the year. The breed is one of the main factors that affect seminal characteristics in goats. In this study, the two Spanish breeds did not differ significantly in the volume and concentration of the ejaculates, although GOT/AST, protein and SP testosterone, plasma testosterone concentrations and scrotal perimeter, differed between the breeds. P bucks had significantly longer scrotal perimeters than did MG bucks, which reflect differences in the size and weight of the breed. These breed differences are similar to that reported by Karagiannidis *et al*. [20], who found significant differences among breeds in almost all the seminal characteristics that they examined in the Alpine and Damascus breeds, or Pérez and Mateos [21] comparing Malagueña and Verata bucks, and in mixed-breed goat bucks [22].

Photoperiod had a significant effect on scrotum size, particularly in autumn under the natural photoperiod. Reaction times did not differ significantly between breeds, or between photoperiod treatments. It has been reported [23] an average reaction time of 98 s in Creole bucks. Probably, the long-term training of the bucks in our study (starting at 4-6 months of age), and the weekly collection of ejaculates mitigated any possible external effects on reaction times.

In our study and others [1], plasma testosterone concentrations were lowest in winter and autumn, highest in summer, and relatively low in autumn, showing a well-defined seasonal pattern, which differed significantly between breeds. The absence of an effect of artificial photoperiod on plasma testosterone concentrations confirms that the secretion of testosterone is markedly seasonal with, such that alternating photoperiod regimes cannot modify it.

In the present experiment, breed, photoperiod, and season had a significant effect on SP protein content, which suggests that might have an effect on the metabolics of animals and, consequently, on the biochemical components of SP. Unlike plasma levels, SP testosterone concentrations were highest in winter, a phenomenon observed in Markhoz goats [24].

In our study, GOT/AST differed significantly between breeds and seasons. That variable has been rarely studied in small ruminants and can be a useful indicator of testicular activity.

## Conclusions

Under the conditions in our study, semen concentrations were highest in spring and summer, and plasma testosterone concentrations were highest in summer. SP testosterone concentrations were highest in winter, and the concentrations of the GOT/AST complex were lowest in spring. Recognition of those seasonal and breed-specific differences in the performance of bucks should help to improve the management of individual semen samples for use in AI programs.

## Authors’ Contributions

FA and JAA designed the experiment and FA carried out the experiment. The article was drafted by JAA and revision was made by FA. All authors have read and approved the final manuscript.

## References

[1] Delgadillo J.A, Chemineau P (1992). Abolition of the seasonal release of luteinizing hormone and testosterone in alpine male goats (*Capra hircus*) by short photoperiodic cycles. J. Reprod. Fert.

[2] Caballero I, Parrilla I, Alminana C, del Olmo D, Roca J, Martinez E.A, Vazquez J.M (2012). Seminal plasma proteins as modulators of the sperm function and their application in sperm biotechnologies. Reprod. Domes. Anim.

[3] Juyena N.S, Stelletta C (2012). Seminal plasma: An essential attribute to spermatozoa. J. Androl.

[4] Pérez-Pé R, Cebrián-Pérez J.A, Muino-Blanco T (2001). Semen plasma proteins prevent cold-shock membrane damage to ram spermatozoa. Theriogenology.

[5] Kumar P, Yadav B, Yadav S (2013). Effect of zinc and selenium supplementation on antioxidative status of seminal plasma and testosterone, T-4 and T-3 level in goat blood serum. J. Appl. Anim. Res.

[6] Wang W, Luo J, Sun S, Xi L, Gao Q, Haile A.B, Shi H, Zhang W, Shi H (2015). The effect of season on spermatozoa motility, plasma membrane and acrosome integrity in fresh and frozen-thawed semen from Xinong Saanen bucks. Reprod. Domes. Anim.

[7] Xu Z.Z, McDonald M.F, McCutcheon S.N, Blair H.T (1991). Seasonal variation in testis size, gonadotrophin secretion and pituitary responsiveness to GnRH in rams of two breeds differing in time of onset of the breeding season. Anim Reprod. Sci.

[8] Casao A, Cebrian I, Asumpcao M, Perez-Pe R, Abecia J, Forcada F, Cebrian-Perez J, Muino-Blanco T (2010). Seasonal variations of melatonin in ram seminal plasma are correlated to those of testosterone and antioxidant enzymes. Reprod. Biol. Endocrinol.

[9] Casao A, Pérez-Pé R, Abecia J.A, Forcada F, Muino-Blanco M.T, Cebrián-Pérez J.A (2013). The effect of exogenous melatonin during the non-reproductive season on the seminal plasma hormonal profile and the antioxidant defence system of Rasa aragonesa rams. Anim. Reprod. Sci.

[10] LaFalci V.S.N, Tortorella H, Rodrigues J.L, Brandelli A (2002). Seasonal variation of goat seminal plasma proteins. Theriogenology.

[11] Ramachandran N, Singh N.P, Ranjan R, Singh M.K, Shinde A.K (2016). Assessment of rearing systems and seasons on nutrient intake and semen freezability in Jamunapari bucks. Indian J. Anim. Sci.

[12] Kumar N, Rai B, Bhat S.A, Kharche S.D, Gangwar C, Jindal S.K, Chandra S (2016). Effect of management system and season on semen freezability in Jakhrana bucks. Vet. World.

[13] Delgadillo J.A, Leboeuf B, Chemineau P (1991). Decrease in the seasonality of sexual behavior and sperm production in bucks by exposure to short photoperiodic cycles. Theriogenology.

[14] Leboeuf B, Furstoss V, Guillouet P, Boué P (2004). Production of semen for artificial insemination from alpine and saanen bucks under different photoperiodic cycles. S. Afr. J. Anim. Sci.

[15] Santiago-Moreno J, Toledano-Díaz A, Castaño C, Coloma M.A, Esteso M.C, Prieto M.T, Delgadillo J.A, López-Sebastián A (2013). Photoperiod and melatonin treatments for controlling sperm parameters, testicular and accessory sex glands size in male Iberian ibex: A model for captive mountain ruminants. Anim. Reprod. Sci.

[16] Cooper T.G, Brazil C, Swan S.H, Overstreet J.W (2007). Ejaculate volume is seriously underestimated when semen is pipetted or decanted into cylinders from the collection vessel. J. Androl.

[17] Delgadillo J.A, Carrillo E, Moran J, Duarte G, Chemineau P, Malpaux B (2001). Induction of sexual activity of 1 male creole goats in subtropical northern Mexico using long days and melatonin. J. Anim. Sci.

[18] Jeyendran R.S, Van der Ven H.H, Perez-Pelaez M, Crabo B.G, Zaneveld L.J (1984). Development of an assay to assess the functional integrity of the human sperm membrane and its relationship to other semen characteristics. J. Reprod. Fertil.

[19] Zarazaga L.A, Guzmán J.L, Domínguez C, Pérez M.C, Prieto R (2009). Effects of season and feeding level on reproductive activity and semen quality in Payoya buck goats. Theriogenology.

[20] Karagiannidis A, Varsakeli S, Karatzas G (2000). Characteristics and seasonal variations in the semen of alpine, saanen and damascus goat bucks born and raised in Greece. Theriogenology.

[21] Pérez B, Mateos E (1996). Effect of photoperiod on semen production and quality in bucks of Verata and Malaguena breeds. Small Rumin. Res.

[22] (2015). Ángel-García, O., Meza-Herrera, C.A., Guillen-Muñoz, J.M., Carrillo-Castellanos, E., Luna-Orozco, J.R., Mellado, M., Véliz-Deras, F.G. (. J. Appl. Anim. Res.

[23] Chemineau P, Varo H, Grudé A (1986). Sexual behaviour and gonadal activity during the year in the tropical Creole meat goat. II. Male mating behaviour, testis diameter, ejaculate characteristics and fertility. Reprod. Nutr. Devel.

[24] Farshad A, Yousefi A, Moghaddam A, Khalili B (2012). Seasonal changes in serum testosterone, LDH concentration and semen characteristics in Markhoz goats. Asian Aust. J. Anim. Sci.

